# Electronic, Magnetic, and Optical Properties of Metal Adsorbed g-ZnO Systems

**DOI:** 10.3389/fchem.2022.943902

**Published:** 2022-06-30

**Authors:** Yang Shen, Zhihao Yuan, Zhen Cui, Deming Ma, Kunqi Yang, Yanbo Dong, Fangping Wang, Ai Du, Enling Li

**Affiliations:** ^1^ School of Science, Xi’an University of Technology, Xi’an, China; ^2^ School of Automation and Information Engineering, Xi’an University of Technology, Xi’an, China; ^3^ Shanghai Key Laboratory of Special Artificial Microstructure Materials and Technology, School of Physics Science and Engineering, Tongji University, Shanghai, China

**Keywords:** g-ZnO, magnetism, main group metal, transition metal, first-principles

## Abstract

2D ZnO is one of the most attractive materials for potential applications in photocatalysis, gas and light detection, ultraviolet light-emitting diodes, resistive memory, and pressure-sensitive devices. The electronic structures, magnetic properties, and optical properties of M (Li, Na, Mg, Ca, or Ga) and TM (Cr, Co, Cu, Ag, or Au) adsorbed g-ZnO were investigated with density functional theory (DFT). It is found that the band structure, charge density difference, electron spin density, work function, and absorption spectrum of g-ZnO can be tuned by adsorbing M or TM atoms. More specifically, the specific charge transfer occurs between g-ZnO and adsorbed atom, indicating the formation of a covalent bond. The work functions of M adsorbed g-ZnO systems are obviously smaller than that of intrinsic g-ZnO, implying great potential in high-efficiency field emission devices. The Li, Na, Mg, Ca, Ga, Ag, or Au adsorbed g-ZnO systems, the Cr adsorbed g-ZnO system, and the Co or Cu adsorbed g-ZnO systems exhibit non-magnetic semiconductor proprieties, magnetic semiconductor proprieties, and magnetic metal proprieties, respectively. In addition, the magnetic moments of Cr, Co, or Cu adsorbed g-ZnO systems are 4 *μ*_B_, 3 *μ*_B_, or 1 *μ*_B_, respectively, which are mainly derived from adsorbed atoms, suggesting potential applications in nano-scale spintronics devices. Compared with the TM absorbed g-ZnO systems, the M adsorbed g-ZnO systems have more obvious absorption peaks for visible light, particularly for Mg or Ca adsorbed g-ZnO systems. Their absorption peaks appear in the near-infrared region, suggesting great potential in solar photocatalysis. Our work contributes to the design and fabrication of high-efficiency field emission devices, nano-scale spintronics devices, and visible-light responsive photocatalytic materials.

## Introduction

The discovery of graphene (Novoselov et al., 2004) has stimulated research into other two-dimensional (2D) materials, such as transition metal dichalcogenides (TMDCs: MoS_2_, WSe_2_, ReS_2_, PtSe_2_, and NbSe_2_), black and blue scales (Zhu and Tománek, 2014; Li et al., 2015; Zhao et al., 2017; Sun and Schwingenschlogl, 2020; Sun et al., 2021), silica, and transition metal oxides (TMOs) (Sahin et al., 2009; Sun and Schwingenschlögl, 2021a; Chen et al., 2021; Lv et al., 2021). Compared with three-dimensional (3D) bulk and wafer materials, 2D materials exhibit superior electron transport, optics, mechanics, and magnetic properties (Gong et al., 2017; Tan et al., 2017), which have been applied in the fields of gas sensing (Zhang et al., 2010; Ziletti et al., 2015; Mahabal et al., 2016; Kooti et al., 2019; Sun et al., 2019), photocatalytic devices (Sun et al., 2017a; Wang et al., 2018a; Cui et al., 2020a), spintronic devices (Yuan et al., 2013; Sun et al., 2017b; Sun et al., 2018a; Cao et al., 2018) and piezoelectric devices (Komsa et al., 2012; Pospischil et al., 2014; Ross et al., 2014; He et al., 2015; Sun et al., 2017c; Sun et al., 2018b; Cui et al., 2020b; Cui et al., 2020c; Cui et al., 2021a; Cui et al., 2021b; Sun and Schwingenschlögl, 2021b).

As one of the II-VI direct bandgap semiconductor materials, ZnO exhibits the characteristics of a wide bandgap, strong radiation resistance, and high exciton binding energy, whose bandgap is about 3.37 eV and the exciton binding energy is up to 60 meV at room temperature (Tusche et al., 2007). ZnO exhibits piezoelectric effect, high chemical stability, high electrochemical coupling coefficient (Weirum et al., 2010), high activity, environmental friendliness, and low acquisition cost, therefore owning significant application potential in the fields of ultraviolet laser emitters, gas, and light detection (Pan et al., 2014; Sahoo et al., 2016; Zhang and Cui, 2022a), as well as photocatalysis (Guan et al., 2017; Guan et al., 2018).

Recently,Claeyssens et al. (2005) predicted the stable existence of graphene-like zinc oxide (g-ZnO). Zhang et al. (2014) report that B, N, or C doped g-ZnO exhibits strong chemisorption for CO. Wang et al. (Cui et al., 2019) conducted a mixed density functional study on the effects of rotation angle and biaxial strain on g-ZnO/TMDCs heterojunctions. In addition to doping and building heterojunctions, adsorption (Zhang and Cui, 2022b) is another efficient way to modify 2D materials. Cui et al. (Wang et al., 2018b; Cui et al., 2020d; Cui et al., 2021c) demonstrated the possibility of reducing the work function of g-GaN adsorption and increasing the absorption of visible light via absorbing transition metals (TMs). Guan et al. (2020) found that the adsorption of transition metal atoms onto graphene with extended-line defects induces magnetism and spin polarization. Wang et al. (2014) found that V, Cr, Fe, Co, Cu, Sc, or Mn absorbed MoS_2_ showed magnetism. Chen et al. (2019) found that Cr, Mn, Fe, Co, or Cu absorbed g-GaN exhibited magnetism. Meanwhile, Zhao et al. (2013) successfully prepared graphene/ZnO composites and applied them to adsorb Cu (II), Pb (II) and Cr (III) in aqueous solution. Luo et al. (2017) synthesized g-ZnO nanosheets and used hybridization density functional theory to calculate cation-anion passivation co-doped g-ZnO for the design of efficient aqueous redox photocatalysts. According to our current knowledge, there are relatively few detailed reports on the adsorption of g-ZnO systems by M (Li, Na, Mg, Ca, or Ga) and TM (Cr, Co, Cu, Ag, or Au). The electronic structure, magnetic and optical properties of the g-ZnO after adsorption require more in-depth exploration.

Here, the electronic, magnetic, and optical properties of M (Li, Na, Mg, Ca, or Ga) and TM (Cr, Co, Cu, Ag, or Au) adsorbed g-ZnO systems were studied using the first-principles based on DFT. The band structure, charge density difference, electron spin density, work function, magnetic properties, and absorption spectrum of each system were analyzed, respectively. The results provide a theoretical basis for the design and fabrication of high-efficiency field emission devices, nano-scale spintronics devices, and visible-light responsive photocatalytic materials.

## Calculation Methods and Models

**FIGURE 1:**
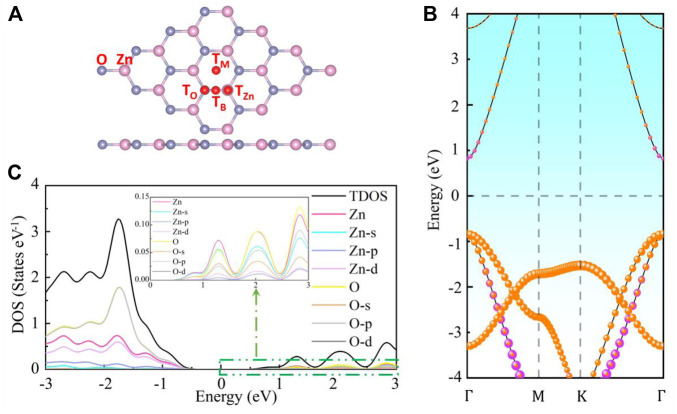
The **(A)** crystal structure, **(B)** energy band structure, and **(C)** density of state of intrinsic g-ZnO.

The electronic, magnetic and optical properties of M (Li, Na, Mg, Ca, or Ga) and TM (Cr, Co, Cu, Ag, or Au) adsorbed g-ZnO systems are calculated by adopting the first principles based on DFT. The electron exchange-correlation effects between electrons are treated using the generalized gradient approximation (GGA) in the Perdew-Burke-Ernzerhof (PBE) formula. Weak intermolecular dispersive forces are treated with Grimme’s DFT-D3 method. The cutoff energy, the K point sampling in the Brillouin zone, the mechanical convergence standard, and the energy change of the atoms are set as 500 eV, 3 × 3 × 1, 0.01 eV Å^−1^, and 10^−5^ eV, respectively. The model of g-ZnO is a 4 × 4 × 1 supercell, as displayed in Figure 1A.

Four different stable adsorption sites are named as T_Zn_ (above the Zn atom), T_O_ (above the O atom), T_B_ (above the middle of the Zn-O bond), and T_M_ (above the center of the hexagonal). The vacuum layer (20 Å in thickness) is added to reduce the interaction between periodic adjacent layers. All systems are geometrically optimized before calculating for obtaining the stable equilibrium state, which is judged by the adsorption energy (*E*_ad_) calculated as follows:

$$E_{\text{ad}}=E_{\text{total}}-E_{\text{g}-\text{ZnO}}-\mu_{\text{M/TM}}$$ (1)

where *E*_ad_ represents the adsorption energy, *E*_total_, *E*_g-ZnO_, and *μ*_M/TM_ are the total energy of M or TM adsorbed g-ZnO systems, intrinsic g-ZnO, and the chemical potential of adsorbed atoms, respectively. The Bader charge method is carried out for accurately calculating the charge transfer. The spin-polarized charge density (*ρ* = *ρ*_spin-up_ *-ρ*_spin-down_) of the Cr, Co, or Cu adsorbed g-ZnO systems are calculated.

## Results and Discussions

The energy band structure of intrinsic g-ZnO is shown in Figure 1B, which demonstrates that it is a direct semiconductor. The total density of state (TDOS) and the density of states for the contribution of electrons in different orbits are shown in Figure 1C. The adsorption energy (*E*_ad_), charge transfer (*C*), magnetic moment (*M*_total_), bandgap (*E*_g_), and adsorption height (*D*) of the M or TM absorbed g-ZnO systems are listed in Table 1.

**TABLE 1:** The adsorption energy (*E*_ad_), charge transfer (*C*), magnetic moment (*M*_total_), bandgap (*E*_g_), and adsorption height (*D*) of the M or TM absorbed g-ZnO systems.

Type	Atom	Adsorption sites	*E*_ad_ (eV)	*C* (∣e∣)	*M*_total_ (*μ*_B_)	*E*_g_ (eV)	*D* (Å)
M	Li	T_M_	−3.374	−0.868	0	0	0.979
	Na	T_M_	−2.283	−0.814	0	0	1.559
	Mg	T_M_	−2.975	−1.340	0	0.613	1.065
	Ca	T_M_	−5.971	−1.478	0	0.908	1.259
	Ga	T_Zn_	−4.203	−0.680	0	0	1.433
TM	Cr	T_O_	−3.161	−0.360	4	2.068	1.865
	Co	T_M_	−2.683	−0.254	3	0.511	1.897
	Cu	T_M_	−1.499	−0.093	1	1.970	1.867
	Ag	T_M_	−0.946	−0.111	0	2.011	2.139
	Au	T_O_	−1.395	+0.127	0	1.982	2.019

**FIGURE 2:**
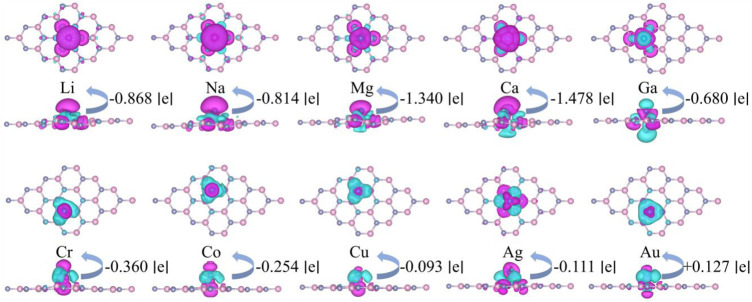
The differential charge density of M or TM adsorbed g-ZnO systems. The blue area and purple area represent electron aggregation and electron dissipation, respectively. And the iso-value is set as 5 × 10^−4^ e Å^−3^.

It is shown that the most stable adsorption sites and the adsorption heights of each system are both different. All systems are slightly deformed due to the interatomic interaction, as shown in Figure 2. Their charge differential densities (CDD) are calculated as follows:

$$△\rho =\rho_{\text{total}}-\rho_{\text{ZnO}}-\rho_{\text{M/TM}}$$ (2)

where *ρ*_total_, *ρ*_ZnO_, and *ρ*_M/TM_ presents the charge densities of M or TM adsorbed g-ZnO systems, intrinsic g-ZnO, and M or TM atoms, respectively. The differential charge density of M or TM adsorbed g-ZnO systems are illustrated in Figure 2, where the specific charge transfer occurred between ZnO and M or TM, which indicates the formation of a covalent bond between ZnO and M or TM atoms.

**FIGURE 3:**
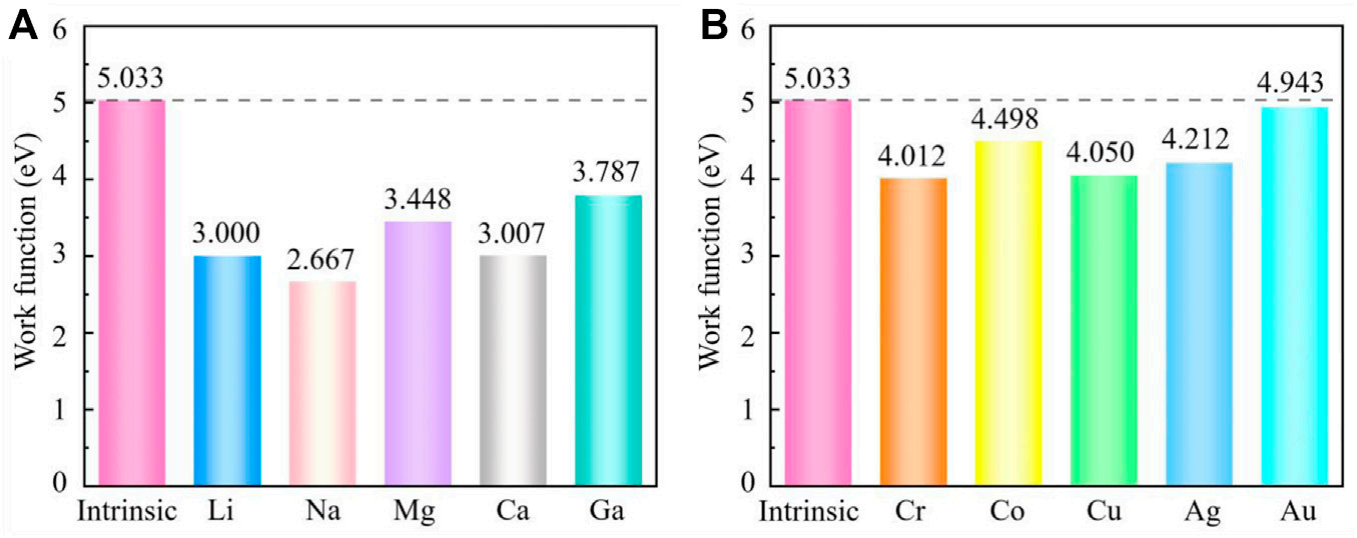
The work functions of the intrinsic g-ZnO and **(A)** M or **(B)** TM adsorbed g-ZnO systems.

The redistribution of charge leads to the creation of a dipole moment, which causes a change in the work function. The work functions are shown in Figure 3. Especially, Na adsorbed g-ZnO system has the lowest work function of 2.667 eV, about 47% lower than that of intrinsic g-ZnO. All adsorption systems have lower work functions than the intrinsic g-ZnO. At the same time, M adsorbed g-ZnO systems have much lower work functions than those of TM adsorbed g-ZnO systems, indicating that M adsorbed g-ZnO systems have strong field emission capabilities.

**FIGURE 4:**
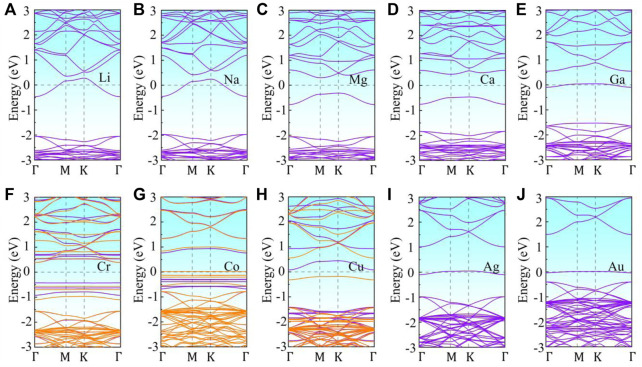
The band structures of M or TM adsorbed g-ZnO systems: **(A)** Li, **(B)** Na, **(C)** Mg, **(D)** Ca, **(E)** Ga, **(F)** Cr, **(G)** Co, **(H)** Cu, **(I)** Ag, **(J)** Au. Purple line indicates spin up, and orange line denotes spin down. The Fermi level is shifted to zero.

To further investigate the effect of metal adsorption on the electronic properties and magnetic properties of monolayer g-ZnO, the energy band structure of the adsorbed system was calculated. The band structure of M or TM adsorbed g-ZnO systems are shown in Figure 4. The gap of the Ca, Mg, Ni, or Pt adsorbed g-ZnO systems are 0.908 eV, 0.613 eV, 2.497 eV, or 2.560 eV, respectively, exhibiting characteristics of non-magnetic semiconductors. The gap of Cr adsorbed g-ZnO system is 2.068 eV, exhibiting characteristics of a magnetic semiconductor. The gap of the Co or Cu adsorbed g-ZnO systems are 0.511 eV or 1.970 eV, respectively, exhibiting characteristics of magnetic metals.

**FIGURE 5:**
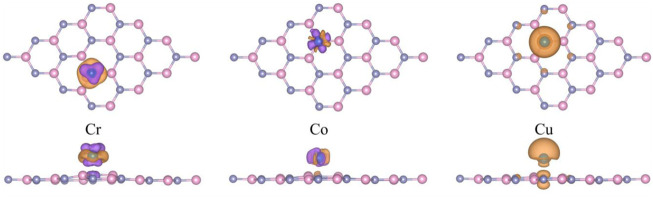
The spin-polarized charge density of Cr, Co, or Cu adsorbed g-ZnO systems. The purple areas represent spin up, and the orange areas represent spin down. And the iso-value is 1 × 10^−3^ e Å^−3^.

For further investigating the derivation mechanism of magnetism, the spin-polarized charge density (*ρ* = *ρ*_spin-up_ *-ρ*_spin-down_) of the Cr, Co, or Cu adsorbed g-ZnO systems are calculated, as shown in Figure 5. The magnetic moments of the Cr, Co, or Cu adsorbed g-ZnO systems are 4 *μ*_B_, 3 *μ*_B_, or 1 *μ*_B_, respectively. Cui et al. reported that the Co and Cu adsorbed Pb_2_Se_3_ systems produced magnetic moments of 0.152 *μ*_B_ and 0.491 *μ*_B_, respectively (Cui et al., 2022). Our results are similar to theirs. From Figure 5, it can be observed that the magnetic moments are mainly derived from adsorbed transition metal atoms. The results suggest the possibility of tuning the magnetic properties of g-ZnO by adsorbing Cr, Co, or Cu as well the potential of applications in nano-scale spintronics devices.

**FIGURE 6:**
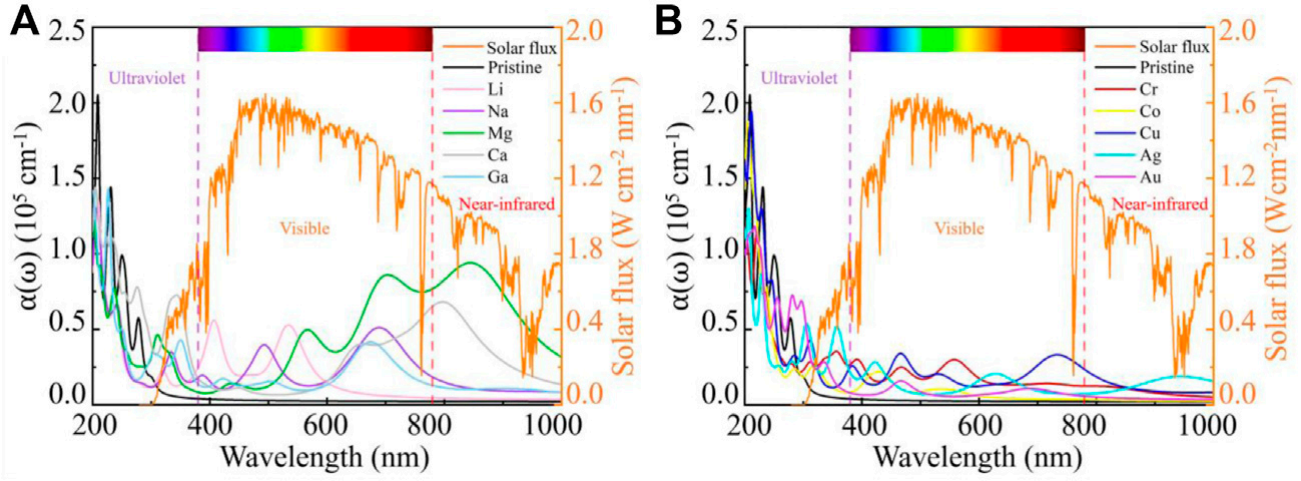
Absorption spectrum of intrinsic g-ZnO and **(A)** M or **(B)** TM adsorbed g-ZnO systems.

The light absorption coefficients [*α*(ω)] of intrinsic g-ZnO and M or TM adsorbed g-ZnO systems are calculated as follows:

$$\alpha \left(\omega \right)=\sqrt{2\omega }{\left[\sqrt{\varepsilon_{1}^{2}\left(\omega \right)+\varepsilon_{2}^{2}\left(\omega \right)-\varepsilon_{1}\left(\omega \right)}\right]}^{\frac{1}{2}}$$ (3)

where *ω*, *ε*_1_(*ω*), and *ε*_2_(*ω*) are the frequency of the photon, real part, and imaginary part of the dielectric constant, respectively. The absorption spectrum of the intrinsic ZnO and metal adsorbed g-ZnO systems are shown in Figure 6. It is shown that the intrinsic g-ZnO has almost no absorption of visible light, while M or TM adsorbed g-ZnO systems have several firm absorption peaks in the visible light region. For example, the absorption peaks of the Li, Na, or Mg adsorbed g-ZnO system are located at 406.6 nm and 534.2 nm, 492.4 nm and 689.2 nm, or 567.6 nm and 704.8 nm, respectively. In addition, the absorption peaks of the Cu adsorbed g-ZnO system are located at 465.1 nm and 733.7 nm. In particular, the *α*(ω) of the Mg adsorbed g-ZnO system is up to 8.633 × 10^4^ cm^−1^ at 704.8 nm. At the same time, Cui et al. (2018) reported the optical properties of g-GaN adsorbed by alkali metals with an absorption peak in the range of 551 nm–708 nm with an absorption coefficient of 2.5 × 10^4^ cm^−1^. Ren et al. (2019) reported a two-dimensional van der Waals heterostructure based on ZnO/Mg(OH)_2_ with an absorption peak of 4.8 × 10^4^ cm^−1^ at 415.75 nm. Xia et al. (2022) reported a two-dimensional GaN/ZnO heterostructure with an absorption peak in the visible range with an intensity of about 2 × 10^4^ cm^−1^. The reports of the above scholars are similar to our results. This indicated significant potential value in visible-light photocatalytic. In addition, the Mg or Ca adsorbed g-ZnO systems have prominent absorption peaks for the near-infrared region, also meaning the potential in solar photocatalysis. Therefore, the optical properties of g-ZnO can be effectively tuned by adsorbing Li, Na, Mg, Ca, or Cu.

In general, compared with the TM absorbed g-ZnO systems, the M adsorbed g-ZnO systems have more obvious absorption peaks for visible light, particularly for Mg or Ca adsorbed g-ZnO systems. Their absorption peaks appear in the near-infrared region, suggesting great potential in solar photocatalysis.

## Conclusion

The electronic, magnetic and optical properties of M (Li, Na, Mg, Ca, or Ga) and TM (Cr, Co, Cu, Ag, or Au) adsorbed g-ZnO systems were studied using the first-principles on DFT. It is found that the band structure, charge density difference, electron spin density, work function, and absorption spectrum of g-ZnO can be tuned by adsorbing M or TM atoms. The work functions of M adsorbed g-ZnO systems are obviously smaller than that of intrinsic g-ZnO, implying great potential in high-efficiency field emission devices. The Li, Na, Mg, Ca, Ga, Ag, or Au adsorbed g-ZnO systems, the Cr adsorbed g-ZnO system, and the Co or Cu adsorbed g-ZnO systems exhibit non-magnetic semiconductor proprieties, magnetic semiconductor proprieties, and magnetic metal proprieties, respectively. In addition, the magnetic moments of Cr, Co, or Cu adsorbed g-ZnO systems are 4 *μ*_B_, 3 *μ*_B_, or 1 *μ*_B_, respectively, which are mainly derived from adsorbed atoms, suggesting potential applications in nano-scale spintronics devices. Compared with the TM absorbed g-ZnO systems, the M adsorbed g-ZnO systems have more obvious absorption peaks for visible light, particularly for Mg or Ca adsorbed g-ZnO systems. Their absorption peaks appear in the near-infrared region, suggesting great potential in solar photocatalysis. Our work contributes to the design and fabrication of high-efficiency field emission devices, nano-scale spintronics devices, and visible-light responsive photocatalytic materials.

## Data Availability

The raw data supporting the conclusions of this article will be made available by the authors, without undue reservation.
